# The Initial Detection of Mycotoxins Released and Accumulated in the Golden Jackal (*Canis aureus*): Investigating the Potential of Carnivores as Environmental Bioindicators

**DOI:** 10.3390/ijms26083755

**Published:** 2025-04-16

**Authors:** Péter Fehér, Zsófia Molnár, Mihály Péter Pálfi, Anikó Pálfiné Lábadi, Patrik Plank, István Lakatos, Miklós Heltai, László Szemethy, Viktor Stéger, Zsuzsanna Szőke

**Affiliations:** 1 Department of Genetics and Genomics, Institute of Genetics and Biotechnology, Hungarian University of Agriculture and Life Sciences, H-2100 Gödöllő, Hungary; feher.peter.arpad@uni-mate.hu; 2 Agribiotechnology and Precision Breeding for Food Security National Laboratory, Department of Animal Biotechnology, Institute of Genetics and Biotechnology, Hungarian University of Agriculture and Life Sciences, H-2100 Gödöllő, Hungary; molnar.zsofia@uni-mate.hu (Z.M.); plankpatrik94@gmail.com (P.P.); istvan.lakatos@am.gov.hu (I.L.); 3 Zsanai Hunting Association, H-6411 Zsana, Hungary; palfimihaly@gmail.com (M.P.P.); labadianiko@gmail.com (A.P.L.); 4 Department of Regional Game Management, Ministry of Agriculture, H-1052 Budapest, Hungary; 5 Department of Wildlife Biology and Management, Institute for Wildlife Management and Nature Conservation, Hungarian University of Agriculture and Life Sciences, H-2100 Gödöllő, Hungary; heltai.miklos.gabor@uni-mate.hu; 6 Institute of Biology, University of Pécs, H-7426 Pécs, Hungary; szemethy.laszlo@pte.hu

**Keywords:** mycotoxins, aflatoxins, deoxynivalenol, fumonisin B1, ochratoxin-A, zearalenone, malondialdehyde, golden jackal, carnivore

## Abstract

This study investigated the presence and levels of five key mycotoxins—aflatoxins (AFs), deoxynivalenol (DON), fumonisin B1 (FB1), ochratoxin-A (OTA), and zearalenone (ZEN) and its metabolite alpha-zearalenol (α-ZOL)—in liver samples from 19 golden jackals (*Canis aureus*) in southern Hungary. Golden jackals, as apex predators with a diverse diet encompassing both plant and animal matter, can serve as valuable bioindicators of environmental mycotoxin contamination. Genetic analysis confirmed the canid samples as coming from golden jackals, excluding the possibility of domestic dogs or hybrid individuals. All samples tested positive for at least three mycotoxins, with multiple mycotoxins frequently co-occurring. DON was detected in 95% of the samples, followed by FB1 (79%) and ZEN (42%). ZOL, AFs, and OTA were present in all samples. Significant differences in mycotoxin concentrations were observed between age groups and sexes for some mycotoxins. Specifically, adult males exhibited higher ZEN concentrations, adult females had higher OTA levels, and females generally showed significantly higher DON concentrations than males. For all investigated individuals, we found significantly higher concentrations of ZEN, alpha-ZOL, and OTA in adult samples. Malondialdehyde (MDA), an indicator of oxidative stress, was also measured and correlated with mycotoxin levels. Pareto analysis suggested a correlation between MDA and OTA/ZEN. These findings highlight the exposure of golden jackals to a range of mycotoxins in their natural environments, potentially through both plant and animal food sources, and underscore the potential of these canids as sentinels for mycotoxin contamination in ecosystems.

## 1. Introduction

Rising temperatures, land and water scarcity, and extreme weather events have caused unprecedented damage to food systems. These variables and extreme events can introduce or exacerbate several food safety and security hazards worldwide, with major consequences for public health and international trade [1]. Changing temperature and precipitation patterns have led to modifications in the geographical distribution and endurance of foodborne pathogens [2]. One of the most significant emerging hazards is the increasing occurrence of mycotoxins. Mycotoxins are secondary metabolites produced by a wide range of filamentous fungi. Consumption of such contaminated food and feed can cause harmful effects on both human and animal health, including hepatotoxicity, nephrotoxicity, neurotoxicity, immunotoxicity, and carcinogenicity. Mycotoxins can harm reproduction and increase mortality too [3]. The most important mycotoxigenic fungi are members of the *Aspergillus*, *Fusarium*, and *Penicillium* genera [4], with aflatoxins (AF), deoxynivalenol (DON), fumonisins (FUMs), ochratoxins (OTA), and zearalenone (ZEN) being the most relevant mycotoxins [5]. These toxins account for worldwide losses of millions of dollars annually in human and animal health, and are condemned for agricultural products [6]. Nephrocarcinogenic compounds have mainly been found in cereals and fruit products, such as dried fruits and grape juice. They enter the kidneys, liver, and blood of farm animals through transfer from animal feed [7,8].

Ochratoxin A (OTA), another potent mycotoxin, primarily affects reproductive and developmental systems. Studies in rats have shown that continuous dietary exposure to OTA causes post-implantation foetal toxicity, delays puberty, and disrupts reproductive health in offspring. Males exhibit reduced testosterone levels and impaired sperm quality, whereas females develop abnormal ovarian follicles. These findings underscore the heightened susceptibility of developing reproductive systems to OTA, compared with those of adults.

Zearalenone (ZEN), produced by *Fusarium* species, acts as a mycoestrogen, owing to its structural similarity to 17β-estradiol, enabling it to bind oestrogen receptors. This endocrine disruptor causes reproductive disorders, such as infertility, hormonal imbalances, and reproductive tract hyperplasia, in livestock, such as pigs and cattle [9,10], and in dogs [11]. Females are especially prone to uterine and ovarian abnormalities [12,13,14], whereas males experience reduced testosterone levels and sperm quality [11]. Beyond reproductive effects, ZEN is linked to immunotoxicity, hepatotoxicity, and genotoxicity [15,16,17]. Alpha-zearalenol, a metabolite of zearalenone, has been detected in liver samples in several studies. The prevalence of alpha-ZOL in the liver could indicate a potential oestrogenic effect of this metabolite, especially given its higher oestrogenic potency than the parent compound zearalenone [18]. Interestingly, the metabolism of ZEN to alpha-zearalenol varies among species [19].

Deoxynivalenol (DON), a mycotoxin produced by *Fusarium graminearum*, inhibits protein synthesis by binding to ribosomes, leading to gastrointestinal issues such as vomiting, weight loss, and appetite suppression [20,21,22]. It also disrupts reproductive health by altering hormone production and steroidogenesis in males [23,24].

Fumonisin B1 (FB1), produced by *Fusarium verticillioides*, contaminates maize and other crops, inducing oxidative stress, apoptosis, and cytokine dysregulation [25,26,27]. Prenatal or postnatal exposure weakens bones, causing thinning of the bone walls [28,29]. FB1 also impairs spermatogenesis and reduces sperm quality in rabbits, boars, and horses [30,31,32].

Moulds can produce different mycotoxins simultaneously, and raw materials can be contaminated with various fungal species simultaneously [33,34]. According to a European study, 75–100% of feed samples contained more than one mycotoxin, which can affect animal health even at low doses [35,36,37,38]. Toxins can interact with each other, functioning both synergistically and antagonistically, thereby enhancing or inhibiting each other. The interaction can be influenced by the age of the animal, the species being studied, the concentration of mycotoxins, and the duration of the interaction [39].

Oxidative stress is associated with the development of several chronic diseases and plays a crucial role in the ageing process [40,41]. Among the various biological targets of oxidative stress, lipids are the most affected class of biomolecules. Lipid oxidation produces several secondary products, primarily aldehydes, which can intensify oxidative damage [42]. Malondialdehyde (MDA) levels in the liver can be affected by various mycotoxins, including DON, ZEN, AFs, and OTA. These mycotoxins have been shown to induce oxidative stress and lipid peroxidation in hepatic tissues [43,44,45].

These mycotoxins are linked to illnesses in humans [46] and domestic animals [47], and may cause acute (e.g., vomiting) or chronic illnesses, such as immune suppression and organ failure [47]. These toxins have been analysed and detected in several wild animals, such as zearalenone in wild boar (*Sus scrofa*) in Italy [48], and aflatoxin in rheas (*Struthio americanus*) in Brazil [49]. The reproduction of these animals could potentially be impacted if they are exposed to contaminated food sources; however, only few data are available on the effects of mycotoxins in canids. As either pet dogs or even working dogs have a lifespan of more than 10 years, longer than that of most farm animals, the quality of dog food is of particular importance in its production. Plant-based ingredients make up a significant proportion of dry dog food, of which grains already require increased quality control due to the risk of mycotoxin contamination [50].

Human–wildlife conflicts are particularly prone to occurring when animals start associating humans with food, especially in places where food is regularly and predictably available [51]. Such areas often lead to higher contact rates among foraging wildlife, thereby increasing the likelihood of disease transmission [52]. In such cases, mycotoxins (T-2 toxin, ochratoxin-A, and zearalenone) have often been detected in piles of urban compost that coyotes (*Canis latrans*) visit to feed [53]. Pollock et al. (2020) [54] found that grizzly bears living near the Canadian Pacific Railway are exposed to both heavy metals and mycotoxins in their natural habitat. These contaminants have been measured in dandelions (*Taraxacum officinale*) growing adjacent to the railway and in grain spilled from hopper cars.

The mycotoxins majorly found in dog food are aflatoxin B1, ochratoxin-A, fumonisin B1, zearalenone, and deoxynivalenol, which can cause significant health damage to dogs [55]. As mycotoxins are known to have negative physiological effects in dogs, and hybridisation of golden jackals with dogs has already proved the close taxonomic relationship between dogs and jackals [56,57], we suppose similar negative effects of mycotoxins on golden jackals too. Species identification of individuals using genetic testing is important to avoid mismatch between the two species and to filter out hybrids. Murray et al. (2016) [53] studied the feeding habits of coyotes—the closest relatives of golden jackals in North America—in urban compost sites in Canada. Compost piles from which the coyotes fed were analysed for mycotoxins, and mycotoxins were detected in 86% of the piles, often at concentrations higher than the legal limits for animal feed. The toxins detected in coyote feed were T-2 toxin, ochratoxin A, and Zearalenone.

The golden jackal (*Canis aureus*) is a widespread opportunistic mid-sized canid distributed throughout southern Asia, the Middle East, and southeastern and central Europe [58,59]. Its diet consists mainly of animals, often supplemented with plants [60,61,62]. In human-dominated landscapes, golden jackals can consume discarded animal waste in areas where animal waste and rodent pests are not properly regulated [63]. In Hungary, the main food categories of golden jackals are rodents, big game, and vegetative/plant parts [64,65,66,67]. In the southwestern part of Hungary, Lanszki and Heltai (2002) [64] determined that 46% of the golden jackal’s diet is of plant origin. Lange et al. (2021) [61] found that the summer diet of golden jackals in Croatia is dominated by plant-based food, primarily undigested fruits, vegetables, seeds, branches, grasses, and dried leaves. Penezić and Ćirović (2015) [62] also confirm that both juvenile and adult individuals take advantage of easily accessible food sources, including fruits, sunflower (*Helianthus annuus*), maize (*Zea mays*), and grass (*Poa* sp.). Lanszki et al. (2015) [68] observed that in an area of intensive big game management, golden jackals’ primary food in every season was viscera and carrion of wild ungulates. As the golden jackal is the largest carnivore species in the area and preys on variable prey, we consider it an apex predator in this ecosystem. Apex predators can function as reliable biodiversity indicators [69,70,71]. Golden jackals can consume food items from natural sources [72] and humans; game managers often provide additional food for wild animals, such as with supplemental feeders, as used in this study. Plant-based food containing mycotoxins can therefore enter the jackal’s body directly (e.g., by consuming winter supplementary feed) and indirectly through a short food chain (grain–small rodent–jackal; winter supplementary feed–prey–jackal). Consequently, we can suppose that golden jackals can serve as a bioindicator of mycotoxin occurrence in the Carpathian Basin’s ecosystem. High levels and negative physiological effects of mycotoxins in cervids have already been identified In Hungary [73]. Mycotoxins have been shown to be transmitted through the food chain [74]. Therefore, the main objectives of our study were to reveal the emergence and, if so, the levels of five mycotoxins (aflatoxin, deoxynivalenol, fumonisin B1, ochratoxin-A, and zearalenone) in genetically pure-blooded golden jackals in Hungary. Additionally, we analysed the mycotoxin concentrations in the main food item, the corn (*Zea mays*) on game feeders, and the level of MDA in the liver, to reveal possible oxidative stress.

## 2. Results

### 2.1. Genetic Analysis

**Figure 1:**
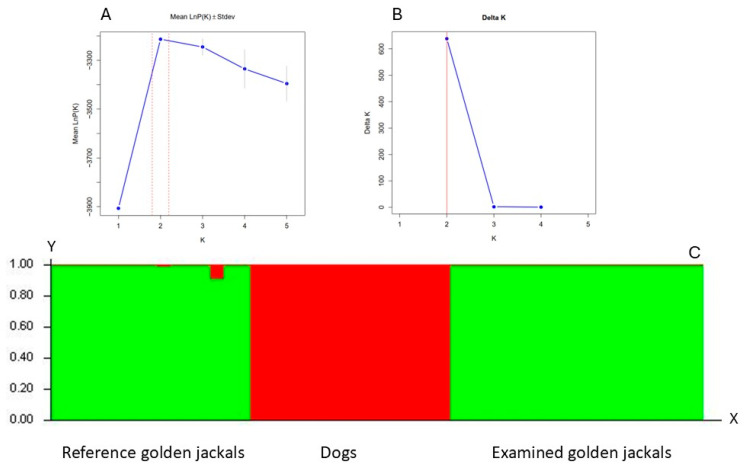
The results of the Bayesian clustering of examined jackal samples and reference dog and golden jackal samples. (**A**) The value of the average log-likelihood (mean Ln P(K)) could be used to infer which cluster number (K) was the most appropriate for clustering the data. (**B**) The probability of the models according to cluster size based on the second-order rate of change in log-likelihood values (Delta K). (**C**) Bar plot of membership probabilities from K = 2. On the y-axis are the likelihood values, which show the probability of assigning each individual to different populations. The values on the x-axis generally represent the individuals belonging to the populations analysed.

Bayesian clustering analysis detected the highest average likelihood scores for two genetic clusters (K = 2) in the dataset, where reference dog and golden jackal samples were separated into two different groups, and the examined samples were genetically the same as the reference jackal samples, without hybridisation (Figure 1).

### 2.2. Mycotoxins and Malondialdehyde

ZOL, AFs, and OTA were present in all samples, ZEN was present in 42% of the samples (n = 8), FB1 in 79% of the samples (n = 15), and DON in 95% of the samples (n = 18). All mycotoxins investigated were found in 31.6% of the samples (n = 6), five mycotoxins were found in 57.8% of the samples (n = 11), and three and four mycotoxins were found in 5.3–5.3% of the samples (n = 1–1), respectively (Table 1).

For ZEN, alpha-ZOL, and OTA, significantly higher values were obtained in the adult age group, regardless of sex. In the case of ZEN, the level of significance was z = −2.64, *p* < 0.01; in the case of alpha-ZOL, it was z = 2.12, *p* < 0.05; and in the case of OTA, the level of significance was Z = 2.66, *p* < 0.01 (Table 2).

We found several significant differences in concentrations between the age groups (Table 3). The ZEN concentration was significantly higher (*p* < 0.05) in adult males than in juvenile males. Although the SD was very big and the sample size was relatively low, the mean ZEN concentration, and also the maximum, were onefold higher in adult males. The OTA concentration was significantly higher (*p* < 0.05) in adult females than in juvenile ones, but the differences in the mean, median, and maximum values were much lower than in the previous case. For the other toxins (α-ZOL, AF, FB1, and DON), we did not find significant differences between age groups. The concentration of malondialdehyde was significantly higher (*p* < 0.05) in juvenile than adult males.

Comparing the mycotoxin concentrations between sexes, we found a significant difference in the case of DON only, where the mean, median, and maximum concentrations were twice as big in females than males (t = −3.77, *p* < 0.01). For the other mycotoxins and MDA, the differences were not significant (Table 4).

All maize samples utilised for feeding game species contained detectable levels of aflatoxin, deoxynivalenol, ochratoxin-A, and fumonisin B1. Zearalenone was not detectable in sample1, and only minimal quantities were detectable in sample2. For the other mycotoxins, the maximum permitted levels were not exceeded (Table 5).

Searching for correlations between mycotoxins, we found a moderate positive correlation between ZEN and OTA (*p* < 0.01), and we detected a moderate negative correlation in the case of ZEN and MDA (*p* < 0.01). However, no significant relationships were found for the other mycotoxins (Table 6).

**Figure 2:**
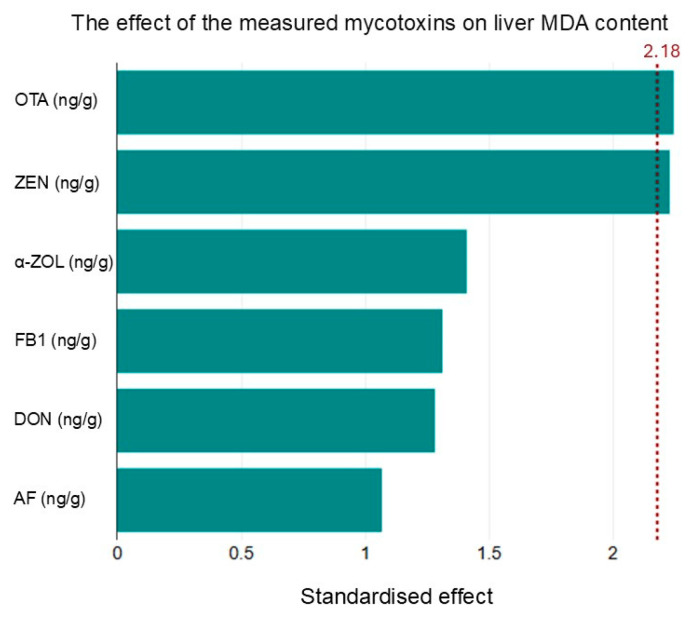
The impact of detected mycotoxins on liver MDA levels. Pareto analysis was used to determine the effect of mycotoxin concentration on MDA concentration in the liver.

The Pareto analysis suggested that in golden jackals, the MDA concentration was most significantly influenced by OTA and ZEN (Figure 2). However, this was not confirmed by the correlation analysis, since there was no significant correlation between MDA and OTA, and there was a significant negative correlation between MDA and ZEN (Figure 2).

## 3. Discussion

Mycotoxins, produced by various moulds, pose significant threats to both human and animal health. These toxins, including aflatoxins, deoxynivalenol (DON), fumonisin B1 (FB1), ochratoxin-A (OTA), and zearalenone (ZEN), can cause a range of health issues, from hepatotoxicity to reproductive disorders. This study on golden jackals highlights their potential as bioindicators for environmental mycotoxin contamination.

We successfully detected all six mycotoxins in golden jackals. Various mycotoxins have also been detected in other wildlife species [48,49,73], and wild animals are exposed to various environmental factors throughout the year [75]. Wild animals provide a good model for studying mycotoxin emergence, levels, and influences. Since wild boars are a suitable species for this topic of study, scientists have studied the presence and effects of several mycotoxins, such as OTA [76,77,78], ZEN [48,78], and AFB_1_ [79]. In a study on the class of birds (Aves), residues of AF and OTA were detected in a few cases in seeds used for feeding wild living birds [80]. Different mycotoxin levels (AFB_1_, DON, ZEN, T-2 toxin, and OTA) in different food samples were also detected in the feed of red-crowned cranes (*Grus japonensis*) in China [81]. The effects of most of these mycotoxins are already known in dogs, a species that is taxonomically closer to the golden jackal; therefore, it can be assumed that the mycotoxins we have studied also show similar symptoms in jackals. Clinical aflatoxicosis in dogs encompasses digestive, neurological, hepatic, nephrotic, and haemodynamic alterations [82]. Zearalenone inclusion in food can induce significant changes in the reproductive system [83]. In canines, DON may induce anorexia, weight loss, emesis, diarrhoea, and potentially gastrointestinal haemorrhage [84]. Although little research is available on the effects of fumonisins on dogs, it is suspected that they may pose hepatotoxic and nephrotoxic risks or cause immunosuppression [85]. Canine intoxication by ochratoxins may manifest as clinical symptoms such as anorexia, excessive thirst, polyuria, polydipsia, anxiety, prostration, restlessness, weakness, and mortality [86]. The detection of multiple mycotoxins in canine liver samples underscores their role in signalling environmental contamination. The presence of DON, FB1, and other mycotoxins in a high percentage of samples suggests widespread exposure through both plant and animal food sources.

In addition to poorly stored plant-based feeds, natural phytocenoses can also contain mycotoxins, which can be transferred to wild animals during feeding. In Argentina, scientists have detected several fungal metabolites, including mycotoxins, in natural grasses (*Poaceae*) intended for grazing cattle. They were able to detect type A trichothecenes, mainly T-2 toxin and HT-2 toxin (both up to 5000 μg/kg), and zearalenone (up to 2000 μg/kg), all at very high frequencies and levels [72]. We also detected golden jackals in the study area that could access grain feed on game feeding grounds (Supplementary Figure S1). A significant proportion of jackal samples (57.9%) did not contain zearalenone, which may be the reason why it was not measurable in maize samples used as winter supplementary feed for ungulates. We can remark that the levels of different mycotoxins show significant variation from year to year, depending on the environmental factors present at a given time. In 2024, AF was the dominant toxin, and the concentration of Fusarium toxins, especially ZEN, was low. Thus, the jackals could not frequently ingest these toxins either directly (by feeding on maize) or indirectly (by feeding on the carcasses of ungulates). Among jackals’ primary animal food sources, several mycotoxins have already been detected in cervids (roe deer and fallow deer) and brown hares in Hungary [73].

Mycotoxins, which are natural food and feed contaminants that are toxic to humans and animals, exhibit sex-dependent responses in terms of their accumulation and effects. Generally, males are more sensitive to mycotoxins than females, and the toxic effects often differ between sexes [87]. This sexual dimorphism in mycotoxin accumulation and response can be attributed to several factors. Our results are not consistent with these results. We did not find any differences between the sexes, except in the case of DON, where the concentration was significantly higher (*p* < 0.01) in females. This may be due to the unequal sample sizes of the male and female groups in this study. Considering age, the ZEN concentration was significantly higher (*p* < 0.05) in adult males, and OTA was significantly higher (*p* < 0.05) in adult females. In this case, the results may be distorted, because for ZEN, 11 of the 19 measured samples had a measurement result of 0. Notably, the importance of accounting for sex-dependent responses is often overlooked in toxicology studies involving mycotoxins [87]. This oversight is problematic because sex differences in exposure, anatomy, physiology, biochemistry, and behaviour are prevalent themes in biology [88]. To address this issue, studies should utilise balanced sex and sex × age designs and analyse data by sex and interactions, rather than simply adjusting for sex [88]. This approach would provide a more comprehensive understanding of mycotoxin accumulation and its effects across sexes in animals.

The correlation between malondialdehyde (MDA) levels and certain mycotoxins indicates oxidative stress as a potential mechanism of mycotoxin-induced toxicity. This relationship highlights the importance of monitoring oxidative stress markers alongside mycotoxin levels to understand their health impacts fully.

Mycotoxin accumulation in animals can vary based on age; younger animals are generally more susceptible to higher levels of toxin residues, and may possess more robust detoxification mechanisms in their livers, which could facilitate more effective breakdown and elimination of mycotoxins than in older individuals [89]. According to Adegbeye et al. (2020) [90], younger livestock often have higher levels of toxin residues in their tissues than older animals. This suggests that age is a significant factor in the accumulation and retention of mycotoxins in animals fed contaminated feed. In contrast, our results do not always support this, as the concentrations of ZEN, alpha-ZOL, and OTA were significantly higher in adult than in juvenile animals. Furthermore, the impact of mycotoxins on animals is not limited to their direct accumulation in tissues. The level of oxidative stress is influenced by the length of contamination, presence of multiple contaminants, synergistic effects, toxin concentrations, age and species of the animal, and stage of production [91]. This indicates that age not only affects toxin accumulation, but also influences the response of animals to mycotoxin exposure in terms of oxidative stress.

Our study is the first to provide data on mycotoxin contamination of golden jackals in Europe. We intended to carry out a brief survey as a first step to reveal the emergence of the most important mycotoxins in a medium-sized apex predator. We hypothesised that the opportunistic predator of the golden jackal could intake mycotoxins from various food sources, and that if it could accumulate specific toxins derived from different-sized prey and even from plants, it could provide more comprehensive information on the presence of mycotoxins in semi-natural forest habitats and complex agricultural habitats. Our supposition was only partially proven. We could detect all the studied mycotoxins in the jackal samples, but on the other hand, we found large individual variance in mycotoxin contamination levels, even among samples from the same area and season. We obtained similar results in our previous studies on other game species [68]. There could be several explanations behind this high individual variability. First, the variability in the recent diets of the individuals could be the main reason. Golden jackals are opportunistic predators and can consume a wide range of food items, from the complete carcasses of small prey, to the viscera of big games, and even plants, especially seeds on game feeders. We hypothesised that variable food sources could decrease individual differences in mycotoxin levels for a longer time if individuals accumulated mycotoxins. As we observed high individual variability, we could not prove this hypothesis. However, it is possible that the individual samples were too far apart in time. Another explanation could be the spatial variability in mycotoxin sources. Although our golden jackal samples were collected from a single hunting estate, it was larger than the home range of one individual. If the mycotoxin sources are unevenly distributed in the area, the individuals could have different access to them. Moreover, there could be explanations based on behaviour. Jackals hunt alone or in family groups during different seasons. The diet of family members could be more similar than that of solitary or roaming hunters. Individual sensitivity to mycotoxins could also increase variability. Considering the possible factors, our sample size was too small, and the sampling period, a whole year, was too long to achieve more homogeneous results. As our samples were provided by the gamekeepers on the hunting estate, and hunting success is unpredictable, it was not possible to collect a large number of individuals and achieve a sex- and age-balanced sample.

Golden jackals, with their diverse diet and position as apex predators, could be ideal candidates as a tool for monitoring mycotoxin exposure in ecosystems, but future research should focus on obtaining larger sample sizes and conducting long-term studies to better understand the specific impacts of sex and age on mycotoxin accumulation. Additionally, developing strategies to reduce mycotoxin contamination in food chains is crucial for maintaining ecosystem balance and ensuring animal health.

## 4. Materials and Methods

### 4.1. Sampling Area

The study area was in the southern part of Hungary, in the Great Plains region. The hunting area is located in Bács-Kiskun county, covering an area of 82.9 km^2^. The region is warm and dry, with an average annual temperature of between 10.5 and 10.7 °C. The annual precipitation is between 550 and 580 mm. The forest cover of the hunting area is about 60%, and is mostly a mixture of grey poplar (*Populus × canescens*), scots pine (*Pinus silvestris*), and black pine (*Pinus nigra*), with a smaller proportion of black locust (*Robinia pseudoacacia*). The remaining 40% consists predominantly of grassland-mowers, with a smaller portion being cropland, where mainly the cereals sorghum and corn are grown. The area is typically characterised by big game management (red deer, fallow deer, roe deer, and wild boar), but for habitat developments, the populations of small game animals (common pheasant, hare) are also stabilising. From the point of view of carnivore populations, the red fox and the golden jackal are the most common, with an average of 70 foxes and 30 golden jackals being harvested annually. The golden jackal density is considered to be high in the region, as the size of the golden jackal bag exceeds the national level by 20%. This study was beyond the scope of the 2010/63/EU Directive, and was carried out with the knowledge and permission of the Institutional Animal Welfare Committee of the Hungarian University of Agricultural and Life Sciences, Szent István Campus (MATE-SzIC/87-1/2024).

A total of 19 golden jackal samples were collected in 2024. The sex distribution of the 19 samples was 12 males and 7 females. Among the males, there were 5 juveniles and 7 adults, while among the females, there were 4 juveniles and 3 adults. To mitigate annual effects, samples were collected within the same year and from the same game management unit. In this region, 20 animals were available for hunting; however, in one instance, the liver of an animal was found to be slightly decomposed, and was therefore unsuitable for analysis.

Liver samples were obtained from free-ranging animals who had been legally shot. Hunted animals were sampled immediately. Samples were stored at −70 °C until the analysis. The age (juvenile or adult), sex (female or male), and body weight were recorded for each individual. In the case of jackals, age was defined first, and conclusions were drawn based on body and skull size. More precisely, dentition and tooth wear provided the right information. When comparing the collected jackals, the approximate body weight limit was 10 kg; it was clear that the teeth of jackals above this weight were significantly thicker and stronger than those of juveniles under 10 kg.

### 4.2. Genetic Analyses

To eliminate the possibility of investigating wild living dogs or golden jackal × dog hybrid individuals, our samples were identified using 22 microsatellite loci (Supplementary Table S1) [92,93,94]. Bayesian clustering analysis was used to identify the individuals using the software Structure ver.2.3.4 [95], using both reference dog (n = 15) and golden jackal (n = 15) samples. The Bayesian clustering method and the Markov Chain Monte Carlo (MCMC) simulation were run, assuming no prior information and using an admixture and independent allele frequency models. The simulation was run with 10 independent runs for each K value ranging from 1 to 5, with a burn-in period of 250,000 iterations and 750,000 replications. We used SructureSelector [96] to determine the number of genetic clusters.

### 4.3. Mycotoxin Analyses

Mycotoxin analysis was performed on liver samples and feed (maize) samples. Aflatoxins (total, B1, B2, G1, G2), deoxynivalenol (DON), fumonisin B1 (FB1), ochratoxin-A (OTA), and zearalenone (ZEN) were measured using ELISA; α-Zearalenol in liver samples (α-ZOL) was analysed by GC-MS.

#### 4.3.1. Zearalenone and Alpha-ZOL Analysis

Liver tissue was homogenised with the FastPrep-24 Classic (MP Biomedicals, Irvine, CA, USA) homogenizer in ice-cold sodium acetate buffer of 50 mM (pH = 4.8), and incubated for 3 h at 37 °C in a shaker with the addition of *Helix pomatia* β-glucuronidase/aryl sulfatase (BGALA-RO, Roche, Basel, Switzerland), according to the manufacturer’s instructions. For zearalenone analyses, the Ridascreen Zearalenone (Art No.: R1401 R-Biopharm, Arnhem, Germany) enzyme immunoassay kits were used. Extraction was performed with a mixture of 70% (*v*/*v*) methanol and water (3:1 *v*/*v*). The extracts were centrifuged at room temperature (RT) at 8000× *g* for 5 min, and the supernatants were collected and diluted with assay buffer. After grinding the feed corn samples, a 70:30 (methanol: water) extraction was performed, followed by a 7× sample dilution with assay buffer. A Trilogy© corn sample was used as an internal standard. The assay was run according to the manufacturer’s instructions. Measurements were acquired and data were analysed with the microplate reader Thermo Multiskan^TM^FC (Waltham, MA, USA), equipped with the SkanIt RE software (version 6.1.1.7). The absorbance was measured at 450 nm, with a 630 nm reference wavelength. The limit of detection (LOD) in the liver was 0.075 ng/g (ppb) [73], and the LOD in corn was 1 ng/g (ppb).

Alpha-ZOL was quantified using the previously reported GC-MS method [97], which was used for golden jackal liver analyses. The LOD value for liver samples was 0.08 ng/(ppb).

#### 4.3.2. OTA Measurements

OTA liver measurements were quantified using the previously validated and reported HPLC-FLD method [98]. OTA was determined using an ELISA kit (TOXI-WATCH Ochratoxin-A ELISA Kit, Cat. Nr.: 3000051, Soft Flow Ltd., Pécs, Hungary), according to the manufacturer’s instructions, and measured in triplicates. The recovery rate was 70.34–87.5%, measured using the OTA-spiked golden jackal liver samples. The LOD value in the case of the jackal liver samples was 0.01 ng/g (ppb).

A Ridascreen OTA Elisa (Art No. R1312, Biopharm, Arnhem, Germany) enzyme immunoassay kit was used for OTA analysis of corn samples. After grinding the corn feed samples, the samples were extracted using a kit containing ECO extractor solution, followed by a 10× sample dilution with assay buffer. A Trilogy© corn sample was used as an internal standard. The assay was performed according to the manufacturer’s instructions. The LOD value for corn samples was 0.25 ng/g (ppb).

#### 4.3.3. Aflatoxins and DON Measurements

The AF and DON contents were determined using Toxi-Watch ELISA kits (Soft Flow Ltd., Pécs, Hungary), which were previously validated for different organs/tissues, according to the manufacturer’s instructions. The samples were measured in triplicate. The recovery was 72.8–89.5% for aflatoxin B1-spiked liver tissue samples and 74.56–94.56% for DON, respectively. In the case of maize, the grinded samples were extracted with 23% ethanol, and diluted with 0.01 M PBS buffer in the case of DON 20×. A Trilogy© corn sample was used as an internal standard. The assay was performed according to the manufacturer’s instructions. The limit of detection (LOD) for Aflatoxin B1 in liver tissue was 0.075 ng/g, while the LOD for aflatoxin in corn was 0.75 ng/g. For deoxynivalenol (DON), the LOD in liver tissue was 1.5 ng/g, and in corn, the LOD was 5 ng/g (ppb).

#### 4.3.4. Fumonisin B1

For fumonisin B1 analysis, we used the EUROPROXIMA Fumonisin (5121FUM) (R-Biopharm, Arnhem, Germany) assay kit. This commercial kit was validated for use with serum and different animal tissues. Before the analyses of the liver samples, we tested the recovery rate of FB1-spiked tissue, which was 70.31–85.14%. The limit of detection (LOD) in liver samples was determined to be 1 ng/g (ppb). Corn samples were subjected to grinding, followed by extraction using an 80:20 methanol:dH_2_O solution. Post-centrifugation, the filtered supernatant was diluted fourfold with a dilution buffer. Analyses were conducted in accordance with the manufacturer’s instructions, and samples were measured in triplicate. The LOD for corn was established at 10 ng/g (ppb).

#### 4.3.5. Malondialdehyde Analyses

The evaluation of lipid peroxidation was executed by quantifying malondialdehyde (MDA) concentrations in liver samples through the Thiobarbituric Acid Reactive Substances Assay. The measurements were conducted utilising a Lipid Peroxidation (MDA) Assay Kit (MAK085, Sigma-Aldrich, Merck KGaA, Darmstadt, Germany). Tissue samples, specifically 10 mg, were homogenised on ice in 300 µL of MDA Lysis Buffer containing 3 µL of BHT. Subsequently, the liver samples were centrifuged at 13,000× *g* for 10 min to eliminate insoluble material. The assay was performed in accordance with the manufacturer’s instructions. During this process, the MDA in the prepared liver tissue sample reacted with thiobarbituric acid (TBA) to form an MDA-TBA complex, which was subsequently quantified via colorimetric analysis. For the colorimetric test, a 0.1 mol/L MDA standard was prepared, and a series of dilutions were created to establish a standard curve. The prepared tissue samples and standards were pipetted into a 96-well plate, and colorimetric measurements were obtained using a Thermo MultiskanTM FC (Waltham, MA, USA) equipped with SkanIt RE software (version 6.1.1.7). Absorbance readings were recorded at a wavelength of 532 nm. The absorbance value of the blank (MQ water) was subtracted to account for background interference. Data analysis was conducted using the SkanIt RE software (version 6.1.1.7).

### 4.4. Statistical Analyses

The assessment of results was performed using DATAtab software (DATAtab Online Statistics Calculator, datatab.net, e.U. Graz, Austria). Continuous variables were compared using an independent *t*-test or the Mann–Whitney test. Comparisons between groups were performed using one-way ANOVA. Linear regression was used to analyse the independent correlated factors. Spearman’s and Pearson’s correlation were used to determine the relationship between values, and multiple logistic regression was used to predict the influence of different variables. *p <*  0.05 was considered to indicate statistical significance.

In the case of ZEN, ZOL, AF, OTA, and FB1, our data deviated significantly from the normal distribution. Therefore, we used the Mann–Whitney U-test to search for differences between age groups within sexes. In the case of DON, the data showed a normal distribution; therefore, we used a *t*-test to search for differences between age groups within sexes.

## 5. Conclusions

This study presents the first documented evidence of the presence and accumulation of mycotoxins in the golden jackal species. The golden jackal (*Canis aureus*) is an opportunistic omnivorous predator that occupies the apex predator niche in the majority of Hungarian ecosystems, particularly as larger predators, such as wolves, lynx, and bears, are predominantly found in the northern mountainous regions of the country [99]. The jackal’s diet is diverse, encompassing both animal and plant matter. Additionally, it consumes a substantial quantity of plant material, including fruits, vegetables, seeds, and grasses. Mycotoxins are toxic secondary metabolites produced by fungi that are present in both plant and animal food sources, and golden jackals may be exposed to mycotoxins through both direct and indirect pathways. Jackals may acquire mycotoxins by consuming contaminated plant material or feeding on mycotoxin-exposed animals. Golden jackals, as opportunistic omnivores, are potentially exposed to mycotoxins through their diverse diet, including contaminated prey (with rumen and intestinal contents), scavenged carcasses, and plant matter (crops and fruits). Deoxynivalenol, aflatoxins, ochratoxins, and fumonisins, found in crops and fruits, pose a potential threat. Their dietary flexibility may increase their exposure risk, particularly in agricultural areas. As an apex predator, the golden jackal serves as a potential bioaccumulator too. We detected all the studied mycotoxins from a relatively small sample, independently of the age and sex of the individuals. Golden jackal hunting is allowed year-round. A high number of individuals are shot or captured annually (e.g., 15,697 in 2023) [100]. Consequently, golden jackals could serve as a suitable subject for investigating the presence and effects of mycotoxins in the environment and as an indicator of mycotoxin presence within this ecosystem. While direct studies are lacking, research on other animals suggests that mycotoxins could negatively impact jackal health by affecting metabolism, immunity, and foraging behaviour.

However, it is important to note that the intentional feeding of jackals is not common practice; thus, their exposure to mycotoxins primarily occurs through natural food sources. There are limited data available regarding mycotoxin contamination in cultivated crops and wild plants; therefore, their role in mycotoxin exposure of jackals requires further investigation. In conclusion, the presence of mycotoxins in golden jackals raises important questions about the health implications for these animals and the ecosystems they inhabit. Further research is needed to explore the long-term effects of mycotoxin exposure on golden jackals and to assess the potential risks to other species within their habitat. This study underscores the importance of continued monitoring and research into environmental contaminants to safeguard wildlife health and ecosystem integrity.

## Figures and Tables

**Table 1:** Descriptive data of the concentrations of the examined mycotoxins (ng/g) and malondialdehyde (nM/mg) by sex and age group. The table contains the frequency, mean, median, standard deviation, and minimum and maximum concentrations. Juv.—juvenile; Ad.—adult, Min.—minimum, Max.—maximum.

Mycotoxin and Malondialdehyde	Sex	Age Group	Freq.	Mean	Median	Std. Deviation	Min.	Max.	Number of Samples Under Limit of Detection
ZEA (ng/g)	Male	Juv.	5	0.03	0	0.06	0	0.14	4
		Ad.	7	0.89	0.23	1.28	0	3.52	2
	Female	Juv.	4	0	0	0	0	0	4
		Ad.	3	0.1	0.11	0.1	0	0.19	1
ZOL (ng/g)	Male	Juv.	5	0.51	0.55	0.23	0.2	0.84	0
		Ad.	7	0.99	0.79	0.58	0.46	1.99	0
	Female	Juv.	4	0.51	0.5	0.2	0.34	0.71	0
		Ad.	3	0.82	0.66	0.47	0.45	1.34	0
AF (ng/g)	Male	Juv.	5	0.41	0.3	0.33	0.21	1	0
		Ad.	7	1.42	0.39	1.9	0.04	4.57	0
	Female	Juv.	4	0.46	0.4	0.37	0.09	0.95	0
		Ad.	3	0.26	0.23	0.17	0.1	0.44	0
DON (ng/g)	Male	Juv.	5	8.76	9.32	6.08	0	15.4	1
		Ad.	7	10.96	11.72	7.61	0.34	19.46	0
	Female	Juv.	4	19.42	21.27	5.67	11.2	23.92	0
		Ad.	3	23.9	22.28	4.21	20.74	28.68	0
OTA (ng/g)	Male	Juv.	5	0.06	0.06	0.02	0.05	0.09	0
		Ad.	7	0.11	0.08	0.1	0.05	0.32	0
	Female	Juv.	4	0.04	0.03	0.01	0.03	0.05	0
		Ad.	3	0.08	0.07	0.01	0.07	0.08	0
FB1 (ng/g)	Male	Juv.	5	25.08	24.91	29.8	0	72.91	2
		Ad.	7	31.09	18.49	25.61	0	74.29	1
	Female	Juv.	4	37.81	39.4	37.1	0	72.45	1
		Ad.	3	64.32	63.78	12.05	52.54	76.63	0
MDA (nM/mg)	Male	Juv.	5	2.56	2.42	0.86	1.4	3.46	0
		Ad.	7	1.4	1.17	0.79	0.64	2.89	0
	Female	Juv.	4	2.2	2.31	0.89	1.17	3.02	0
		Ad.	3	3.45	1.48	3.56	1.31	7.56	0

**Table 2:** Differences between age groups for all samples in the examined mycotoxins and MDA (Mann–Whitney U-test). * *p* < 0.05; ** *p* < 0.01; n.s. non-significant.

Mycotoxin and Malondialdehyde	ZEN		Alpha-ZOL		AF		OTA		FB1		MDA	
	U	Z	U	Z	U	Z	U	Z	U	Z	U	Z
Age-dependent mycotoxin accumulation	16	−2.64 (**)	19	−2.12 (*)	45	0 (n.s.)	12.5	−2.66 (**)	34.5	−0.86 (n.s.)	23	−1.8 (n.s.)

**Table 3:** Comparison of mycotoxin and malondialdehyde levels (ng/g; nM/mg) between age groups within sexes (Mann–Whitney U-test). * *p* < 0.05; n.s.—non-significant.

Mycotoxin and Malondialdehyde	ZEN		ZOL		AF		OTA		FB1		MDA	
	U	Z	U	Z	U	Z	U	Z	U	Z	U	Z
Male adult and juvenile	6	−1.99 (*)	8	−1.54 (n.s.)	15	−0.41 (n.s.)	8.5	−1.47 (n.s.)	15	−0.41 (n.s.)	4	−2.19 (*)
Female adult and juvenile	2	−1.76 (n.s.)	3	−1.07 (n.s.)	4	−0.71 (n.s.)	0	−2.14 (*)	4	−0.71 (n.s.)	6	0 (n.s.)

**Table 4:** Comparison of mycotoxin and malondialdehyde levels (ng/g; nM/mg) between sexes (Mann–Whitney U-test). n.s.—non-significant.

Mycotoxin and Malondialdehyde	ZEN		ZOL		AF		OTA		FB1		MDA	
	U	Z	U	Z	U	Z	U	Z	U	Z	U	Z
Sex	29	−1.22 (n.s.)	32	−0.85 (n.s.)	36.5	−0.47 (n.s.)	22.5	−1.65 (n.s.)	27.5	−1.23 (n.s.)	33	−0.76 (n.s.)

**Table 5:** Fungal toxin detection results of corn feed samples from the study area.

Maize (Feed)	ZEN µg/kg (ppb)	Total Aflatoxins µg/kg (ppb)	DON µg/kg (ppb)	OTA µg/kg (ppb)	FB1 µg/kg (ppb)
Sample1	0	1.53	175.24	0.26	1242
Sample2	1.98	2.7	129.82	0.47	1405

**Table 6:** Correlations between mycotoxin and malondialdehyde levels in the liver tissue. **—*p* < 0.01.

		ZEN (ng/g)	ZOL (ng/g)	AF (ng/g)	DON (ng/g)	OTA (ng/g)	FB1 (ng/g)	MDA (nM/mg)
ZEN (ng/g)	Correlation	1	0.25	0.2	−0.09	0.66	−0.16	−0.63
	*p*-value		0.31	0.42	0.715	0.002 (**)	0.521	0.004 (**)
ZOL (ng/g)	Correlation	0.25	1	0.14	0.08	0.19	0.1	−0.17
	*p*-value	0.31		0.566	0.732	0.424	0.676	0.486
AF (ng/g)	Correlation	0.2	0.14	1	0.23	0.08	0.26	−0.26
	*p*-value	0.42	0.566		0.342	0.741	0.275	0.283
DON (ng/g)	Correlation	−0.09	0.08	0.23	1	−0.22	0.44	−0.03
	*p*-value	0.715	0.732	0.342		0.372	0.058	0.898
OTA (ng/g)	Correlation	0.66	0.19	0.08	−0.22	1	−0.05	−0.27
	*p*-value	0.002 (**)	0.424	0.741	0.372		0.834	0.272
FB1 (ng/g)	Correlation	−0.16	0.1	0.26	0.44	−0.05	1	−0.09
	*p*-value	0.521	0.676	0.275	0.058	0.834		0.712
MDA (nM/mg)	Correlation	−0.63	−0.17	−0.26	−0.03	−0.27	−0.09	1
	*p*-value	0.004 (**)	0.486	0.283	0.898	0.272	0.712	

## Data Availability

The raw data supporting the conclusions of this manuscript will be made available by the authors, without undue reservation, to any qualified researcher.

## Supplementary Materials

The following supporting information can be downloaded at https://www.mdpi.com/article/10.3390/ijms26083755/s1.

## Abbreviations

The following abbreviations are used in this manuscript:

AFs	Aflatoxins
AfB1	Aflatoxin B1
AfB2	Aflatoxin B2
AfG1	Aflatoxin G1
AfG2	Aflatoxin G2
DON	Deoxynivalenol
FB1	Fumonisin B1
OTA	Ochratoxin-A
ZEN	Zearalenone
α-ZOL	Alpha-zearalenol
MDA	Malondyaldehide
PBS	Phosphate-buffered saline
GC-MS	Gas chromatography–mass spectrometry
