# Supplementary material for: The Initial Detection of Mycotoxins Released and Accumulated in the Golden Jackal (Canis aureus): Investigating the Potential of Carnivores as Environmental Bioindicators
Source: Int J Mol Sci. 2025 Apr 16;26(8):3755. doi: 10.3390/ijms26083755 (PMC12027561; doi:10.3390/ijms26083755)
Supplement: Supplementary file 1 [file ijms-26-03755-s001.zip › ijms-3522509-supplementary.pdf]

International Journal of *Molecular Sciences*

MDPI

Supplementary materials

The Initial Detection of Mycotoxins Released and Accumulated in the Golden Jackal (*Canis aureus*): Investigating the Potential of Carnivores as Environmental Bioindicators

Péter Fehér 1†, Zsófia Molnár 2†, Mihály Péter Pálfi 3, Anikó Pálfiné Lábadi 3, Patrik, Plank 2, István Lakatos2,4 , Miklós Heltai5, László Szemethy5, Viktor Stéger1*, Zsuzsanna Szőke2*

[1.] Department of Genetics and Genomics, Institute of Genetics and Biotechnology, Hungarian University of Agriculture and Life Sciences, H-2100 Gödöllő, Hungary, feher.peter.arpad@uni-mate.hu, steger.viktor@uni-mate.hu

[2.] Agribiotechnology and Precision Breeding for Food Security National Laboratory, Department of Animal Biotechnology, Institute of Genetics and Biotechnology, Hungarian University of Agriculture and Life Sciences, H-2100 Gödöllő, Hungary, molnar.zsofia@uni-mate.hu, plankpatrik94@gmail.com, ferenczine.szoke.zsuzsanna@uni-mate.hu

[3] Zsanai Hunting Association, H-6411 Zsana, Hungary, palfimihaly@gmail.com, labadianiko@gmail.com

[4] Department of Regional Game Management, Ministry of Agriculture, H-1052 Budapest, Hungary, istvan.lakatos@am.gov.hu

[5] Department of Wildlife Biology and Management, Institute for Wildlife Management and Nature Conservation, Hungarian University of Agriculture and Life Sciences, H-2100 Gödöllő, Hungary, heltai.miklos.gabor@uni-mate.hu

[6] Institute of Biology, University of Pécs, H-7426 Pécs Ifjúság 6, Hungary, szemethy.laszlo@pte.hu

* Correspondence authors: steger.viktor@uni-mate.hu and ferenczine.szoke.zsuzsanna@uni-mate.hu

† These authors contributed equally to this work.

Supplementary materials Figure S1.: Golden jackal eating supplementary feed at the feeding site for ungulate species (camera trap).

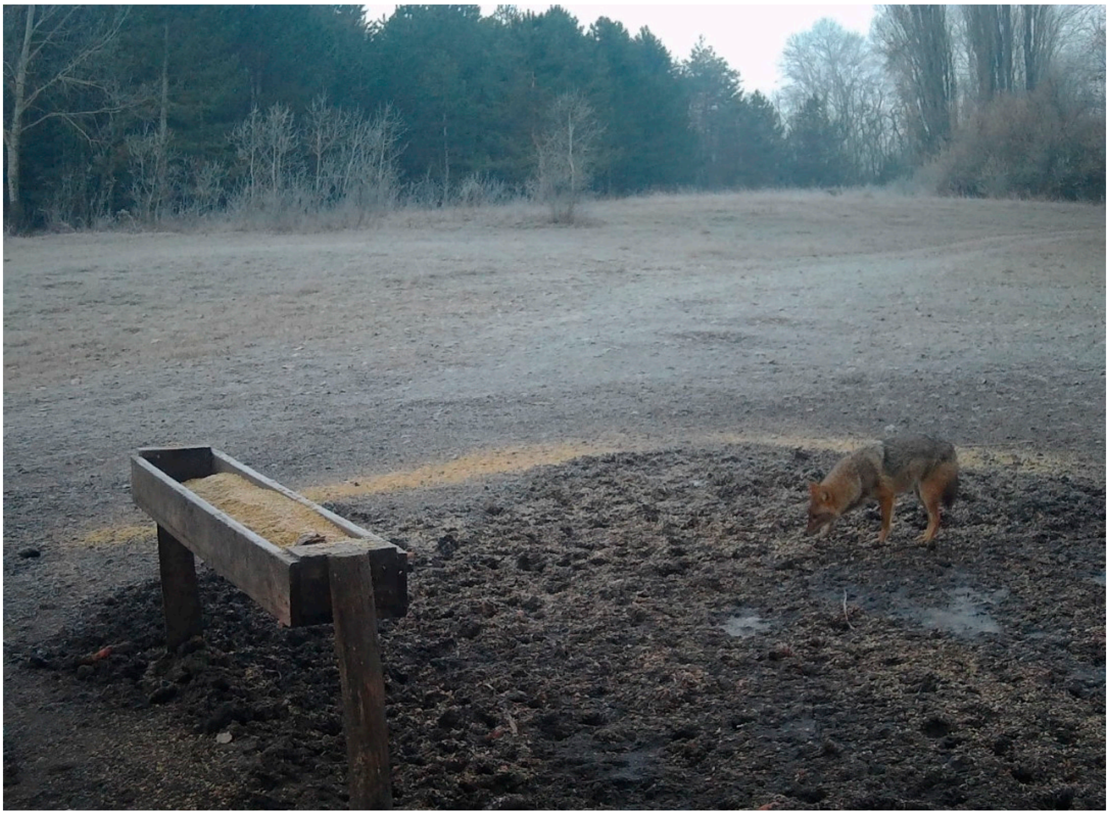

Supplementary materials Table S1.: Description of the genotyped autosomal microsatellite (STR) primers.

Primer name	Forward primer sequnce (5' to 3')	Reverse primer sequence (5' to 3')
AHT137	TACAGAGCTCTTAACTGGGTCC	CCTTGCAAAGTGTCATTGCT
AHTh171	CTCACCAGGCATAGACACTCA	CTCATTTGTTCACGCACCC
AHTh260	CGCTATACCCACACCAGGAC	CCACAGAGGAAGGGATGC
c2001	TCCTCCTCTTCTTTCCATTG	TGAACAGAGTTAAGGATAGACACG
c2054	GCCTTATTCATTGCAGTTAG	ATGCTGAGTTTTGAACTTTCCC
c2096	CCGTCTAAGAGCCTCCC	GACAAGGTTTCCTGGTTCCA
FH2004	CTAAGTGGGGAGCCTCCTCT	ACTGTGACCTACTGAGGTTGCA
FH2010	AATGGAACAGTTGAGCATGCA	CCCCTTACAGCTTCATTTTCCA
FH2088	CCCTCTGCCTACATCTCTGC	TAGGGCATGCATATAACCAGCT
FH2107	CATCATTATTGGGCTCCATGCT	CTGTGTCATCATTAAGCTAAGTCCT
FH2309	GACTGAGTTCTTTCAGCACAGTG	GGCAGCCTTATTATTCATGGAAA
FH2538	CAGAGACAAAGGCTTCCCTG	CCCCTCTACTCCTCCTGCTT
FH3313	TGCACACCCAAAAAGTAAGCC	CAATCTGAAGCCAATCTCATCTCA
FH3377	GAGGAATTAAGAAGATACGAGACC	GGAATACGTTTATTTTCAGCTTGA
PEZ02	TCCTCTCTAACTGCCTATGCTC	GCCCTTGAATATGAACAATGACACT
PEZ05	GCTTCCGGCTCGTATGTTGTG	TTGGGTAACGCCAGGGT
PEZ3	CACTTCTCATACCCAGACTC	CAATATGTCAACTATACTTC
PEZ6	ACACAATTGCATTGTCAAAC	ATGAGCACTGGGTGTTATAC
PEZ8	TATCGACTTTATCACTGTGG	ATGGAGCCTCATGTCTCATC
PEZ11	ATTCTCTGCCTCTCCCTTTG	TGTGGATAATCTCTTCTGTC
PEZ12	GTAGATTAGATCTCAGGCAG	TAGGTCCTGGTAGGGTGTGG
PEZ19	GACTCATGATGTTGTGTATC	TTTGCTCAGTGCTAAGTCTC
